# Changes in presynaptic calcium signalling accompany age‐related deficits in hippocampal LTP and cognitive impairment

**DOI:** 10.1111/acel.13008

**Published:** 2019-07-16

**Authors:** Daniel Pereda, Ibrahim Al‐Osta, Albert E. Okorocha, Alexander Easton, Nicholas A. Hartell

**Affiliations:** ^1^ Department of Neuroscience, Psychology and Behaviour University of Leicester Leicester UK; ^2^ Department of Psychology University of Durham Durham UK; ^3^ Present address: Facultad de Ciencias de la Salud Departamento de Ciencias Médicas Básicas Apartado 456, C/Sta. María Soledad 38200 San Cristóbal de La Laguna Spain; ^4^ Present address: Faculty of pharmacy, Department of Pharmacology and Clinical Pharmacy Elmergib University Alkhoms City Libya; ^5^ Present address: Department of Physiology, Faculty of Basic Medical Sciences Ebonyi State University Ebonyi Nigeria

**Keywords:** aging, CA1, genetically encoded calcium sensor, hippocampus, learning and memory, presynaptic calcium, transgenic mouse, transmitter release

## Abstract

The loss of cognitive function accompanying healthy aging is not associated with extensive or characteristic patterns of cell death, suggesting it is caused by more subtle changes in synaptic properties. In the hippocampal CA1 region, long‐term potentiation requires stronger stimulation for induction in aged rats and mice and long‐term depression becomes more prevalent. An age‐dependent impairment of postsynaptic calcium homeostasis may underpin these effects. We have examined changes in presynaptic calcium signalling in aged mice using a transgenic mouse line (SyG37) that expresses a genetically encoded calcium sensor in presynaptic terminals. SyG37 mice showed an age‐dependent decline in cognitive abilities in behavioural tasks that require hippocampal processing including the Barnes maze, T‐maze and object location but not recognition tests. The incidence of LTP was significantly impaired in animals over 18 months of age. These effects of aging were accompanied by a persistent increase in resting presynaptic calcium, an increase in the presynaptic calcium signal following Schaffer collateral fibre stimulation, an increase in postsynaptic fEPSP slope and a reduction in paired‐pulse facilitation. These effects were not caused by synapse proliferation and were of presynaptic origin since they were evident in single presynaptic boutons. Aged synapses behaved like younger ones when the extracellular calcium concentration was reduced. Raising extracellular calcium had little effect on aged synapses but altered the properties of young synapses into those of their aged counterparts. These effects can be readily explained by an age‐dependent change in the properties or numbers of presynaptic calcium channels.

## 1 INTRODUCTION

Normal aging is associated with a loss in cognitive function. Regions of the brain responsible for learning and memory, including the prefrontal cortex and the hippocampus, are particularly vulnerable. In contrast to age‐related neurodegenerative conditions such as Alzheimer's disease, which is accompanied by extensive cell death and characteristic neuropathological changes (see for example Vinters, 2015), the anatomical changes that accompany the cognitive decline associated with aging are far more subtle.

Within the hippocampus, aging is not associated with a loss of principal cell number (Rapp & Gallagher, 1996), nor is there a consistent reduction in dendritic branching or spine density. Synapse numbers are preserved in the CA1 region of aged rats with spatial learning impairment but there is a reduction in the size of postsynaptic densities of axospinous, perforated synapses (see Burke & Barnes, 2006; Morrison & Baxter, 2012 for reviews). In contrast, in CA3 and parts of the dentate gyrus, there is a decrease in synapse number and synaptophysin immunoreactivity in aged rats with spatial learning deficits. The quantal size of granule cell responses is increased in this region (Foster, Barnes, Rao, & McNaughton, 1991), suggesting amplified synaptic transmission albeit at fewer perforant path‐dentate gyrus synapses.

Hippocampal LTP is disrupted in aged animals with deficits in spatial memory. Induction paradigms that can induce LTP in younger animals are much less effective in the aged brain (Barnes, Rao, & McNaughton, 1996; Deupree, Bradley, & Turner, 1993; Deupree, Turner, & Watters, 1991; Moore, Browning, & Rose, 1993). CA1 neurons show a weaker temporal summation to high‐frequency stimulation, and higher frequencies of stimulation are required for plasticity induction. Aged rats are also more susceptible to LTD and reversal of LTP (Norris, Korol, & Foster, 1996). Age‐related changes in postsynaptic calcium homeostasis may underpin these effects since the direction of plasticity in this region is dependent on the extent to which postsynaptic calcium increases. Buffering intracellular calcium with membrane permeable calcium chelators reduced LTP in young adult rats but enhanced spatial learning and LTP in aged animals (Tonkikh et al., 2006).

The vast majority of studies that have looked at the effects of calcium on aging in the CNS have concentrated on postsynaptic processes primarily because measuring presynaptic calcium in situ is extremely difficult. Any alteration in presynaptic calcium homeostasis would have profound effects on synaptic transmission and plasticity induction. Presynaptic calcium not only triggers transmitter release, the residual calcium signal within the terminal controls release probability and forms of short‐term plasticity including frequency facilitation and paired‐pulse facilitation. These mechanisms are particularly important for shaping responses to bursts of higher frequency such as those that induce LTP through interactions with the postsynaptic cell. Indeed, changes in the concentration of residual presynaptic calcium have been proposed to serve as the mechanism for storage of short‐term memory within neuronal circuits (Mongillo, Barak, & Tsodyks, 2008).

Using a transgenic mouse that expresses a genetically encoded, ratiometric calcium sensor exclusively in presynaptic terminals (Al‐Osta et al., 2018), we have examined the role of presynaptic calcium in synaptic transmission in the CA1 region of the hippocampus in young adult mice and compared how transmission properties and presynaptic calcium signalling are affected in mice with measured cognitive and synaptic deficits associated with age‐related hippocampal dysfunction. We show that aging is accompanied by elevated calcium influx into individual presynaptic terminals and a chronic increase in absolute residual presynaptic calcium that affects transmission properties in the CA1 region of the hippocampus. These mice fail to perform as well as young adult animals in a range of behavioural tasks requiring hippocampal function and brain slices from these animals are less able to undergo LTP. Age‐related changes in residual calcium and associated synaptic properties can be reversed or mimicked by lowering or raising extracellular calcium, respectively. We conclude that age‐dependent changes in homeostatic control of calcium within presynaptic terminals contribute to the synaptic changes and cognitive decline associated with aging.

## 2 RESULTS

### 2.1 Age‐related hippocampal cognitive decline

In order to establish at what age hippocampal function becomes impaired, groups of SyGCaMP2‐mCherry‐positive mice aged up to 6, 12, 18 and 24 months underwent cognitive behavioural tasks which all require various degrees of hippocampal processing. The results are summarized in Figure S1. The T‐maze spontaneous alternation test is highly sensitive to hippocampal dysfunction and a particularly sensitive test of spatial memory in mice (Deacon & Rawlins, 2006). In 6‐ and 12‐month groups, the alternation rates were 87.0 ± 3.0 (*n* = 20) and 86.1 ± 3.2% (*n* = 23), respectively. Values above 80% are expected in strains such as C57BL/6, which is the background strain for our transgenic mice, using this test in the absence of hippocampal dysfunction. The alternation rate dropped significantly in animals that were 18 and 24 months old to 74.1 ± 2.6 (*n* = 29) and 73.6 ± 4.3% (*n* = 11), respectively (*p* < .001; Kruskal–Wallis test). We found significant age‐dependent effects on the performance of mice (Figure S1b; *p* = .0038, 1‐way ANOVA) in the Barnes maze, a nonaversive test of hippocampal‐dependent spatial memory (Barnes, 1979; Fox, Fan, LeVasseur, & Faden, 1998). A multiple comparison analysis revealed that animals 18 or 24 months of age made significantly more mistakes than younger animals (*p* < .05). There was also a clear age‐dependent change in performance in a spontaneous object location (SOL) spatial memory test. Animals aged 18 or 24 months did not show a significant exploration preference for novel combinations of object and location. In contrast, for the spontaneous object recognition (SOR) test, which is independent of the hippocampus (but see Clark, Zola, & Squire, 2000; Forwood, Winters, & Bussey, 2005), mice of all ages were able to recognize objects significantly above chance. Mice also showed a highly statistically significant decrease in burying behaviour with age (*p* < .0005; Kruskal–Wallis test; Figure S1e). This behaviour is dependent on both hippocampus (Deacon, Croucher, & Rawlins, 2002) and prefrontal cortex (Deacon, Penny, & Rawlins, 2003) in C57BL/6 mice. In open field tests, there was no significant effect of age on markers of open field activity with the exception of the number of rearing events within a 5‐min period which also declined with age (Figure S1f). These behavioural data indicate that mice aged 18 months or more are significantly less capable of carrying out tasks that involve spatial memory and which require hippocampal processing.

### 2.2 Impairment of LTP in aged mice

**Figure 1:**
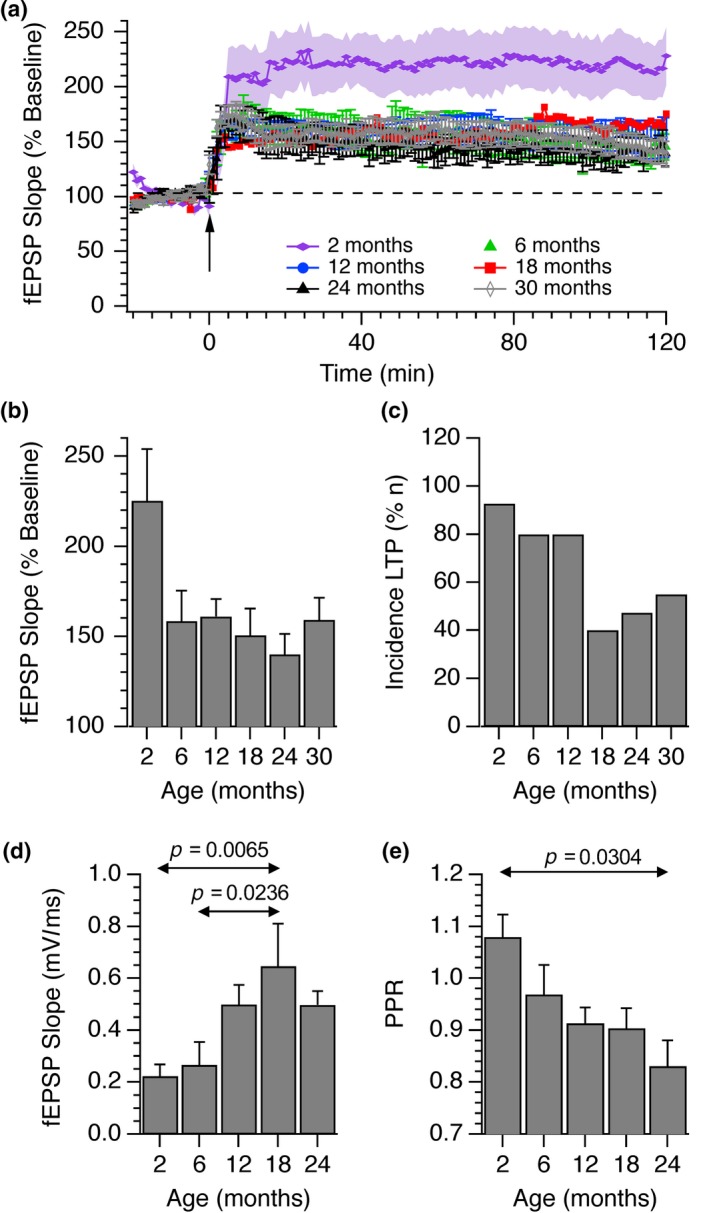
Age‐dependent changes in the incidence of LTP and size of fEPSP responses in the CA1 hippocampus. LTP was induced using a theta burst paradigm in slices obtained from mice of ages between 2 and 30 months. (a) The means and SEMs from *n* = 15 (2 months), *n* = 12 (6 months), *n* = 17 (12 months), *n* = 7 (18 months), *n* = 10 (24 months) and *n* = 12 (30 months) individual experiments are shown. Only those experiments in which a sustained potentiation was observed are shown. (b) The fEPSP mean ± *SEM* initial slopes after 60 min, expressed as a percentage of the pre‐LTP baseline response, are shown. No statistical differences in the extent of the potentiation were observed although there was a general decrease in the amount of potentiation with age (Kruskal–Wallis; *p* = .60). (c) The incidences of experiments where LTP was observed are shown. The total numbers of experiments carried out for each age group from youngest to oldest were *n* = 16, 17, 20, 20, 19 and 20. The mean slopes of fEPSPs (d) and paired‐pulse ratios (e) at each of these age ranges are shown along with standard errors of the mean. 1‐way ANOVA tests revealed statistically significant effects of age on both fEPSP size (*p* = .0026) and PPR (*p* = .046). The *p* values illustrated indicate statistically significant differences between specific age groups (Tukey post hoc test)

**Figure 2:**
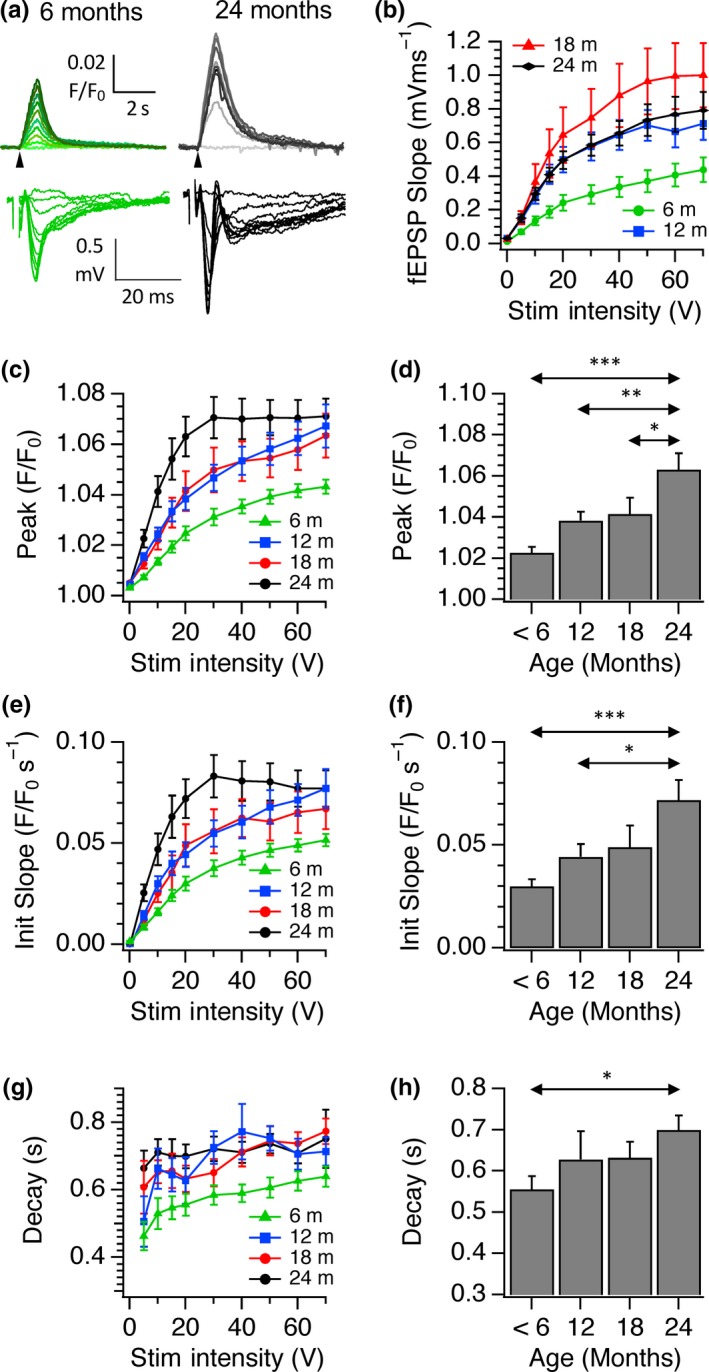
The effect of age on input–output relationships for SyGCaMP2 responses and fEPSPs to electrical stimulation of the SC pathway to CA1. (a) Examples of recordings of SyGCaMP2 fluorescence (*F*/*F*_0_) from 6‐month‐old (green traces) and 24‐month‐old hippocampal slices (greyscale traces). Representative fEPSs are shown underneath. Stimulus intensities were increased from 0 to 70V. (b) Pooled data showing mean ± *SEM* fEPSP slopes recorded from slices taken from groups of animals at age ranges of up to 6 months, 12, 18 and 24 months. Panels (c), (e) and (g) illustrate pooled data of the peak amplitude, initial slope and fast decay time constant of SyGCaMP2 responses. Means and SEMs from *n* = 36 (<6 months), *n* = 16 (12 months), *n* = 13 (18 months) and *n* = 18 (24 months) are shown. Panels (d), (f) and (h) show the mean peak, slope and fast decay values measured at 20 V. Double‐headed arrows indicate where statistical differences were observed and asterisks indicate the level of significance (*p < .05; ** *p* < .01; *** *p* < .001; 1‐way ANOVA with Tukey post hoc test)

We next examined the incidence and amplitude of LTP at SC‐CA1 pyramidal synapses in these mice using a theta burst induction paradigm. Experiments were carried out at each of 6 age groups which were 2, 6, 12, 18, 24 and 30 months. For those experiments where LTP was induced, there was a decline in the amplitude of potentiation with increasing age, measured 60 min after induction, but this was not significant (Figure 1a, b). There was, however, an age‐dependent reduction in the incidence of LTP with age (Figure 2c). Theta burst stimulation‐induced potentiation in 15 out of 16 separate experiments in the 2‐month age group but in only 10 out of 19 experiments at 24 months and 11 out of 20 at 30 months. Thus, the relative incidence dropped from over 90% in young animals to around 50% in older ones.

CA1 field potential responses to fixed intensities of stimulation also increased with age reaching a peak at 18 months (Figure 1d). Paired‐pulse ratios also changed in an age‐dependent manner, suggesting that the increase in response size was a presynaptic effect. Both groups showed statistically significant changes with age (1‐way ANOVA; *p* < 0.05). The arrows and *p* values indicate where significant differences were observed between specific age groups (Tukey post hoc test).

Having established the age range over which we were able to see cognitive decline and alterations in CA1 long‐term and short‐term plasticity, we examined whether calcium signalling in presynaptic boutons was also affected during aging in SyG37 mice. We first characterized responses to Schaffer collateral stimulation to bursts of 20 stimuli delivered at 20 Hz of increasing intensity to examine the input–output relationship of synaptic transmission within this pathway with age. The peak amplitudes, initial slopes and decay time constants of SyGCaMP2 responses were measured and compared. fEPSPs from the same field of view were recorded simultaneously and the slopes of the postsynaptic (N2) component measured. As expected, SyGCaMP2 and fEPSP responses increased with stimulus intensity in all age groups. However, age‐related differences in the peak amplitudes and initial slopes of SyGCaMP2 fluorescence responses were also observed in response to increasing stimulus intensity (Figure 2a, c–f). The amplitudes of CA1 fEPSPs also increased with age although the pattern was slightly different in that the greatest effect occurred in the 18‐month group as opposed to the 24‐month group for the SyGCaMP2 responses (Figure 2a, b).

The ability of cells to maintain calcium levels homeostatically after stimulation was assessed by measuring the decay time constant (tau) of SyGCaMP2 fluorescence recovery. In all age groups, recovery was faster at lower stimulus intensities but there was a gradual slowing of the rate of SyGCaMP2 fluorescence decay with age which was statistically significant between the youngest and oldest groups of animals (Figure 2 g, h). In CA1 therefore, age leads to a steepening of the input–output relationship and an increase in the amount and duration of the calcium signal in the ensemble response of CA1 boutons.

SyGCaMP2 is expressed selectively in excitatory and inhibitory presynaptic terminals (Al‐Osta et al., 2018). The recorded signals within CA1 can originate not only from Schaffer collateral fibre terminals but also from any presynaptic terminals of neurons that are activated synaptically including intrinsic interneurons or CA1 pyramidal cell axon collaterals. To see whether the age‐dependent increase in the size of responses was due to an increase in the relative proportion of the synaptic component of SyGCaMP2 fluorescence, DNQX, picrotoxin and AP5 were used to block AMPA, GABA_A_/Glycine and NMDA receptors, respectively (Figure S2). Blockade of inhibitory and excitatory synaptic transmission completely abolished N2 fEPSPs as expected (Figure S2b, c). In slices obtained from 6‐month‐old animals, SyGCaMP2 responses reduced in amplitude to 51.1 ± 8.5% of control levels, suggesting that ~50% of the total signal originates from presynaptic terminals that have been activated synaptically. This proportion remained statistically unchanged up to 24 months confirming that the age‐dependent increases in SyGCaMP2 responses were not due to a proliferation or long‐term strengthening of synaptic connections but must have occurred at the level of the SC fibres.

#### 2.2.1 Multiphoton measurements of presynaptic calcium from single boutons

**Figure 3:**
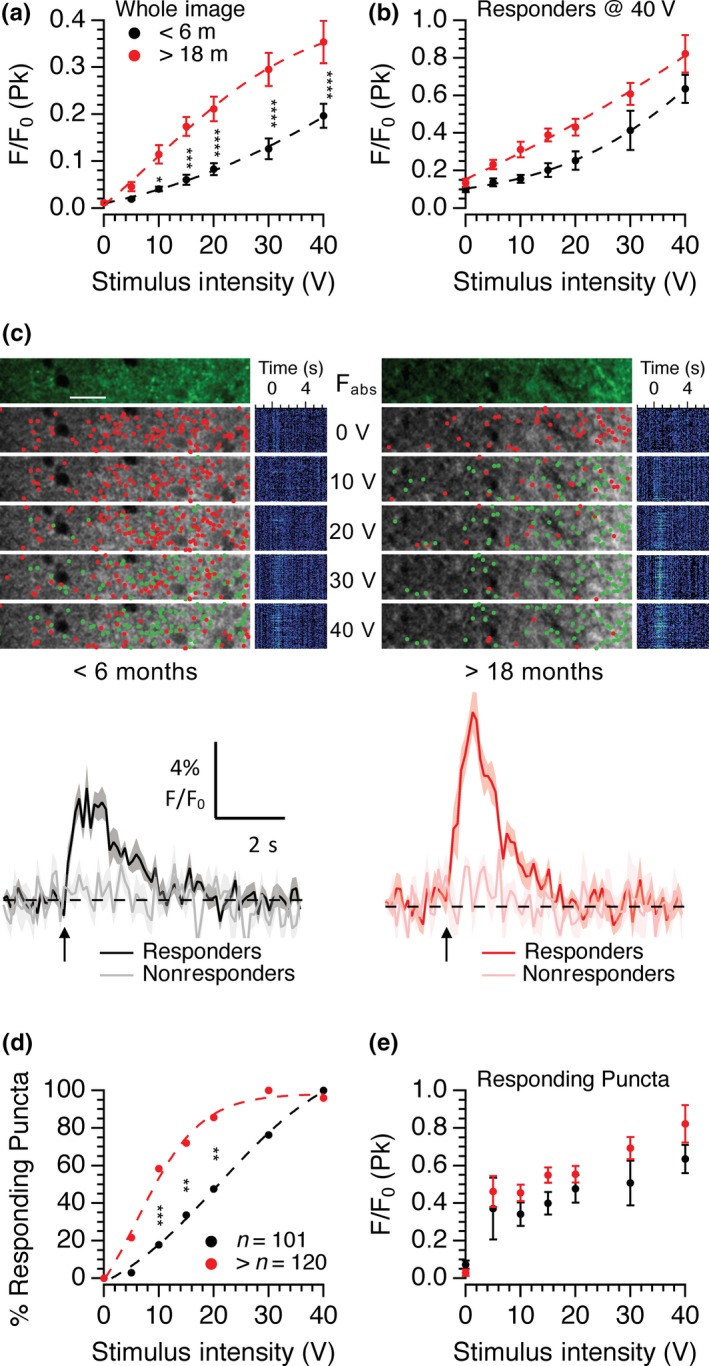
Effects of age on single bouton responses to electrical stimulation. The means and standard errors of whole frame SyGCaMP2 responses to increasing stimulus intensities are shown (a) and for data from identified puncta that responded at 40 V for the entire intensity range (b). Panel (c) shows representative multiphoton images from the *stratum radiatum* of hippocampal slices from 6‐month‐old (left) and 18‐month‐old (right) brain slices. The top image shows the absolute fluorescence before stimulation. Below are images showing the positions of responding (green) and nonresponding (red) puncta. Mean traces from identified single puncta that responded at 40 V are shown underneath for each age group. The lighter traces in each case illustrate responses from background regions which did not respond to stimulation. Black/grey traces were from <6‐month‐old slices and red/pink traces from 18‐month‐old slices. The percentage of puncta that responded at each intensity (d) and the peak responses from only those puncta that responded at each intensity (e) are shown. Data were pooled from slices from 9 (<6 months) and 12 (>18 months) experiments. Two‐way ANOVA with Sidak's multiple comparison test (*p < .05; **p < .01; ***p < .001; ****p < .0001)

Using multiphoton microscopy, it is possible to identify individual synaptic boutons in SyG37 mice and so we next examined whether this age‐dependent increase in SyGCaMP2 fluorescence represents an increase in the amount of calcium mobilized per bouton and/or an increase in the number of boutons recruited. Measurements of background subtracted fluorescence from the full scan area (512 pixels × 64 lines) produced results that were essentially identical to those observed using wide‐field epifluorescence techniques. Responses in aged animals were elicited at lower stimulus intensities than those from young animals and the overall sizes of the responses at a given stimulus intensity were larger (c.f. Figure 2 with Figure 3a). The mean numbers of puncta within fixed scan volumes were 38,100 ± 3,469 per mm^2^ in young animals (*n* = 9 fields of view) and 32,600 ± 2,633 per mm^2^ (*n* = 16) in animals >18 months old. These were not significantly different (*p* = .21; unpaired *t* test), indicating that the densities of boutons expressing SyGCaMP2‐mCherry did not change with age.

Small ROIs were positioned over individual puncta that responded at the maximum stimulus intensity used of 40 V, and then, these same ROIs used to measure responses from images recorded over the entire range of stimulus intensities (Figure 3b). As before, peak mean responses from many ROIs increased with intensity and were consistently larger in slices from aged animals compared to those from young adult mice (Figure 3b). To better establish the cause of this age‐dependent increase in response size, a thresholding method combined with hierarchical clustering was used. Puncta were identified and divided into responding and nonresponding groups at each stimulus intensity, and the positions of these puncta distinguished with green and red dots positioned at the centre of maximum intensity (Figure 3c). The numbers of puncta that responded at each intensity were expressed as a proportion of the total (Figure 3d) along with the mean peak responses of only those puncta that actually gave a measurable response to a given stimulus intensity (Figure 3e).

This method successfully separated responding from nonresponding puncta and revealed that the numbers of responding puncta at each intensity were also different between young and old animals (Figure 3c). In young adult animals, puncta were recruited in a sigmoidal pattern over this intensity range. In old animals, the threshold for recruitment was lower and the rate of recruitment was steeper, reaching a plateau at 30 V. Differences were statistically significant at stimulus intensities at or above 10 V (Figure 3d). Thus, the larger average peak response in older SyG37 animals is, at least in part, due to activation of more puncta at lower intensities. The size of the calcium response per puncta was also significantly increased in slices from aged animals as shown by Figure 3e (*p* = .0023; 2‐way ANOVA aged vs. young). Since only responses from puncta that responded were included in this data set, any effect of age on puncta recruitment is removed. The increase in calcium influx per bouton with age could account for, or contribute to, the apparent lower threshold for detection of responses in single boutons. This is unlikely however because having identified boutons that can respond to stronger stimuli, it should have been possible to detect even a small response from the average of the many boutons recorded at lower stimulus intensities. This was not the case suggesting that the threshold for activation was indeed lower in aged animals.

**Figure 4:**
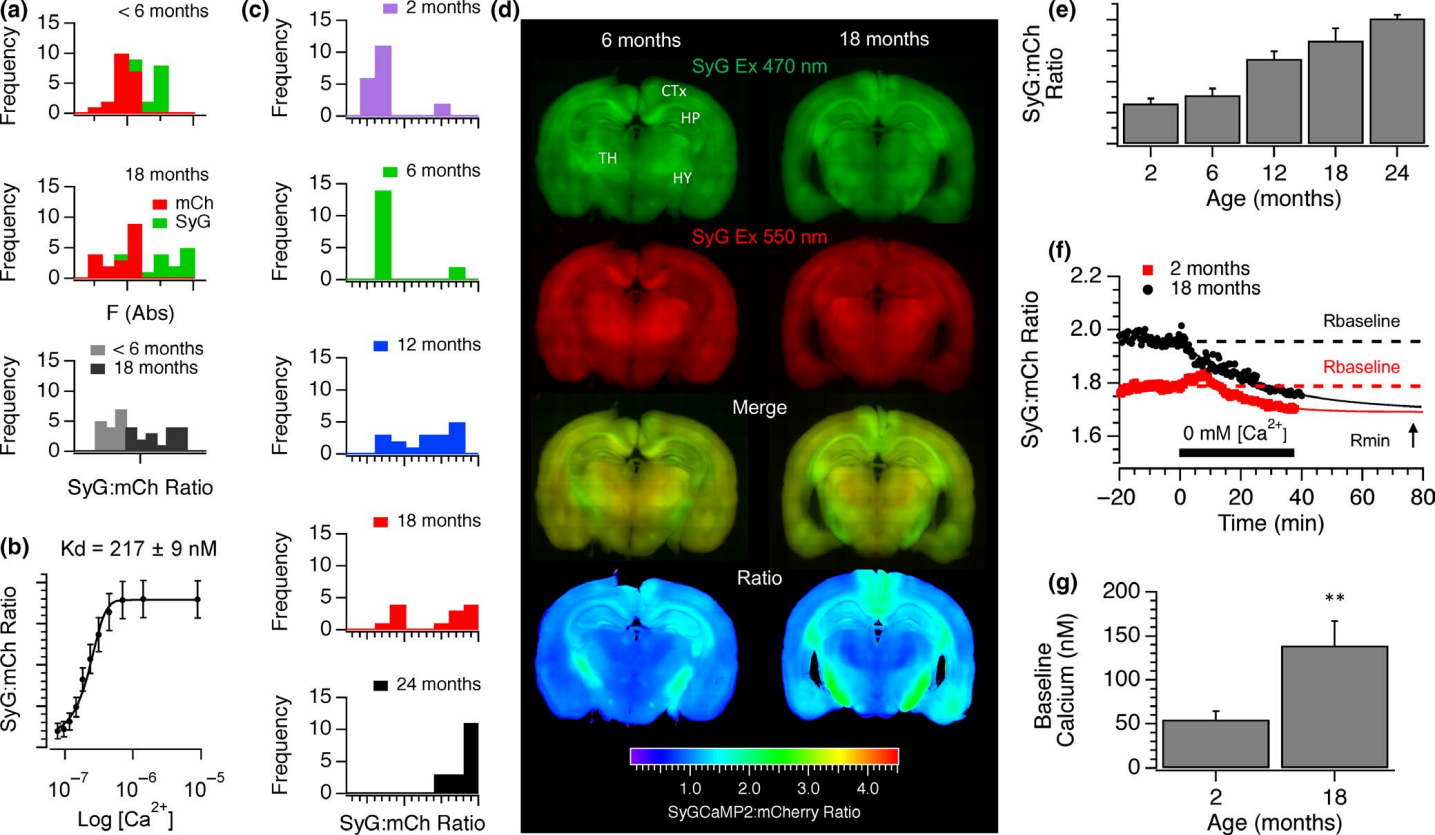
The effects of age on baseline SyGCaMP2 fluorescence and absolute calcium levels. (a) Baseline fluorescence frequency distributions of mCherry (red) and SyGCaMP2 (green) from SyG37 mice aged <6 months (top) and 18 months or more (middle). Shown underneath are the SyG:mCherry ratios for each age group. (b) The relationship between the SyG:mCh ratio of the sensor expressed in SyG37 mice and absolute free calcium measured in permeabilized HEK293 cells. (c) Frequency distributions of the baseline SyG:mCh ratios in 2‐, 6‐, 12‐, 18‐ and 24‐month‐old SyG37 mice. (d) Stitched images of coronal slices from 6‐month‐ and 18‐month‐old mice. SyGCaMP2 and mCherry fluorescence is shown in green and red, respectively. Also shown are merged images and the SyG:mCh ratio after background subtraction using wild‐type, aged matched slices. Images are shown using consistent brightness and contrast settings. (e) Background subtracted SyG:mCh ratios of baseline fluorescence from slices at each age group. Means and standard errors of at least *n* = 12 slices per group are shown. To calibrate baseline calcium, a nominally zero calcium solution was washed on to slices from 2‐month‐ and 18‐month‐old mice in the presence of ionomycin (f). Although the baseline ratio was higher in old slices, the ratio converged to similar values in both sets of mice. Baseline calcium was then calculated for slices from each age group using values of Rmin, Rmax (not shown) and the Kd (panel b). The mean baseline values for 9 young and 7 aged slices are shown in panel (g)

Analysis of the absolute baseline SyGCaMP2 fluorescence revealed a consistent difference between young (<6‐month‐old) and aged (18‐month‐old) animals (Figure 4a). There was no concurrent increase in mCherry fluorescence, suggesting that this was not simply due to increased sensor expression with age. The ratio of SyG:mCh fluorescence compensates for any changes in sensor expression levels. In brain slices prepared from animals under 6 months of age, the SyG:mCh ratio was consistently low compared to the older age group. In permeabilized HEK293 cells expressing the SyGCaMP2‐mCherry sensor, the SyG‐mCh ratio responded to free calcium in a sigmoidal manner and the affinity for free calcium under these conditions was 217 ± 9 nM (Figure 4b). If the ratio values at nominally zero and saturating levels of calcium are known along with the calcium affinity, it is possible to use the SyG:mCh ratio to estimate absolute, residual, presynaptic calcium concentrations.

In brain slices prepared from animals under 2 or 6 months of age, the SyG:mCh ratio was low in almost all slices as shown by plotting the frequency distribution of baseline ratio values. In slices from 12‐month‐old animals, the ratios were distributed more uniformly. In slices from 18‐month‐old animals, the proportion of baseline ratios was clearly split between low and high values but in 24‐month‐old slices, all ratios were in the high range (Figure 4c). This suggests that with age, brain slices gradually transition from having a low average baseline presynaptic calcium concentration to a higher concentration.

We then examined stitched images of SyGCaMP2 and mCherry fluorescence in coronal slices from 6‐ and 18‐month‐old mice to examine whether we could see any spatial differences in basal calcium concentration in CNS with age. Images of mCherry fluorescence revealed the basic distribution of the sensor and show that expression was present throughout the brain but particularly high in the deeper layers of the cortex, the hippocampus, thalamus and fibre tracts, especially those surrounding the hippocampus including the fornix, corpus callosum and fimbria. We have previously shown that within the hippocampus, expression is absent in the cell bodies within the pyramidal cell layer from CA3 to CA1 and subiculum and targeted to both excitatory and inhibitory presynaptic terminals (Al‐Osta et al., 2018). Relative age‐dependent increases in the SyG:mCh ratio were evident in the hippocampus but also notably in the retrosplenial cortex. Age‐dependent increases were observed in the fornix and fimbria, the major input and output pathways to and from the hippocampus (Figure 4d) as well as the postero‐lateral amygdala and pyriform cortex. Within the CA1 region of the hippocampus, the mean SyG:mCh ratio increased with age significantly (Figure 4e).

Nominally zero and saturating calcium solutions were washed onto slices in the presence of the calcium ionophore ionomycin to measure minimum and maximum ratio values (*R*_min_ and *R*_max_), respectively, and absolute baseline calcium in young and old slices measured. The baseline ratios of young and old slices were different as previously demonstrated but upon application of the nominally zero calcium solution, the ratios converged, suggesting that absolute calcium in presynaptic terminals was indeed higher in older slices (Figure 4f). In young slices, baseline calcium was approximately 55 ± 9 nm and in older slices around 139 ± 28 nm (Figure 4g). The values obtained for young slices were very similar to those previously estimated for presynaptic terminals in CA1 from Guinea pigs of a similar age (Wu & Saggau, 1994). The SyG:mCh ratio in SyG37 mice therefore provides a quantitative measurement of absolute baseline presynaptic calcium. We are not aware that absolute resting presynaptic calcium levels have been measured previously in mice but presynaptic calcium levels are raised in aged Fischer 344 rats compared to young adults (Tonkikh et al., 2006).

**Figure 5:**
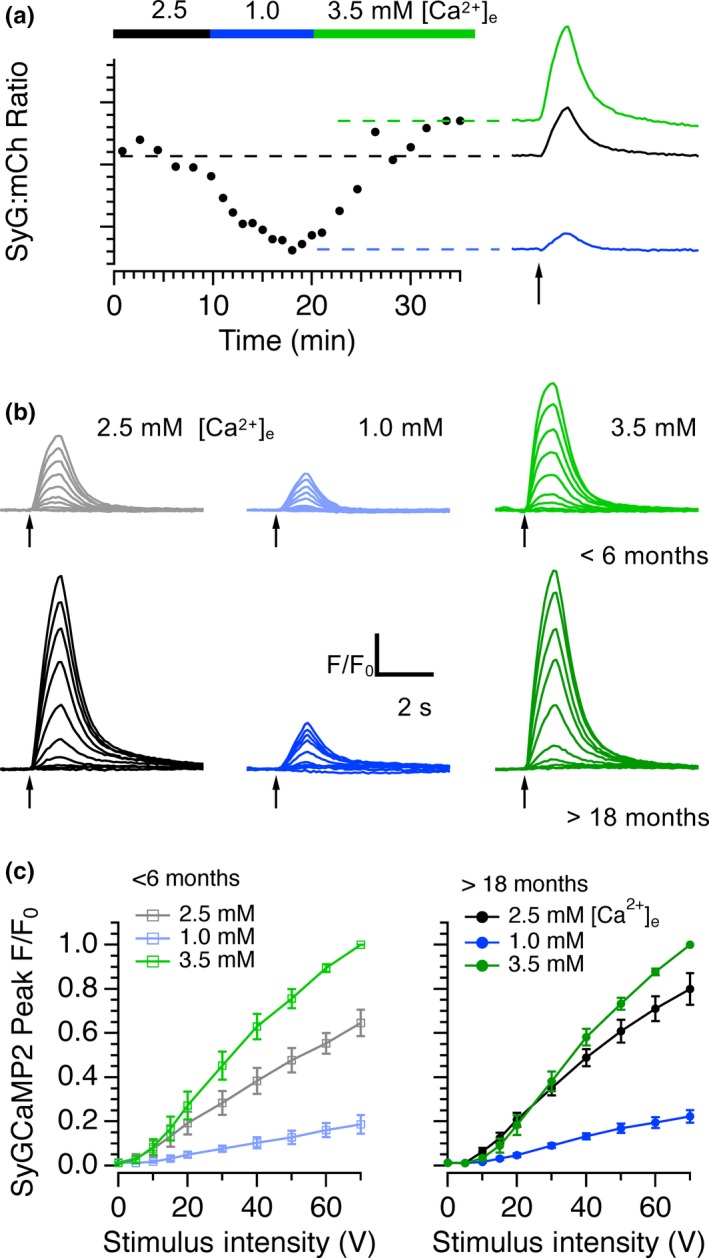
Effects of modulating extracellular calcium on SyGCaMP2 responses in young and aged mice. (a) The baseline SyG:mCherry ratio in a slice from an aged mouse was measured in aCSF containing 2.5 mM extracellular calcium and in modified solutions containing 1 and 3.5 mM calcium. Responses to 20 stimuli at 20Hz are shown under each condition on the right. (b) The effects of stimulus intensity on SyGCaMP2 responses were measured in <6‐month‐old and >18‐month‐old slices in normal, low and high calcium solutions. Pooled data from 6 separate young (left) and aged slices are shown in panel (c) Data were normalized to the peak response measured in 3.5 mM calcium. Raising calcium had a significantly smaller effect on aged slices than on younger slices

The increased size of presynaptic calcium responses to electrical stimulation in aged slices and single boutons suggests an increase in the conductance or density of presynaptic calcium channels. If so, it should be possible to reverse these effects by modifying extracellular calcium concentration. We compared the effects of stimulus intensity in extracellular solutions containing nominally normal (2.5 mM), low (1.0 mM) and high (3.5 mM) extracellular calcium. Lowering extracellular calcium from 2.5 to 1.0 mM reduced the mean CA1 SyG:mCh ratio in slices from aged animals and reduced the amplitudes of responses to electrical stimulation (Figure 5a). The addition of a high extracellular calcium solution (3.5 mM) increased the SyG:mCh ratio to values just greater than those in standard aCSF. It was therefore possible to alter the intra‐terminal calcium concentration by changing the extracellular calcium concentration. In slices from young animals (<6 months of age), lowering the extracellular calcium concentration reduced the size of SyGCaMP2 responses to different intensities of stimulation as expected. In the presence of 3.5 mM [Ca^2+^]_e_, responses increased in size well above those recorded in standard aCSF (Figure 5b, c). In slices from older animals, and consistent with our earlier observations, SyGCaMP2 peak responses were larger than those of younger animals in standard aCSF (Figure 5b). Responses were dramatically reduced in 1.0 mM [Ca^2+^]_e_ but in 3.5 mM [Ca^2+^]_e_, responses increased to levels only slightly higher than those recorded in normal aCSF (Figure 5d). In the aged group, there was no significant difference between responses in high and normal aCSF but there was a clear difference in responses within the young group. Older terminals can be made to behave more like younger ones if the extracellular calcium concentration is reduced. Conversely, young terminals can be made to behave like older terminals by raising extracellular calcium.

## 3 DISCUSSION

We initially established the age ranges over which cognitive decline occurred in our transgenic mice. For behavioural tests that involve spatial memory, and which require an intact hippocampus, we found statistically significant reductions in performance in animals aged for 18 months or more. These results are entirely consistent with studies carried out previously in both rats (Mizoguchi, Shoji, Tanaka, Maruyama, & Tabira, 2009; Robitsek, Fortin, Koh, Gallagher, & Eichenbaum, 2008) and mice (Barreto, Huang, & Giffard, 2010; Ennaceur, Michalikova, van Rensburg, & Chazot, 2008; Forster et al., 1996). Similar age‐related differences were observed in the marble burying test which is dependent on both the hippocampus and prefrontal cortex (Deacon et al., 2002). We also observed an age‐dependent change in spontaneous object location tests; animals up to the age of 12 months were able to distinguish between the locations of objects. Unexpectedly, animals showed a statistically significant preference for the object in the familiar location rather than the novel location. Nevertheless, this preference was indicative of a learned spatial memory and was absent in animals older than 18 months of age. In an object recognition test, animals of all age groups were able to distinguish between familiar and novel objects, suggesting that spatial memory, as opposed to novel object recognition, is affected by age. SyG37 mice aged 18 months or more are therefore significantly less capable of carrying out tasks that involve spatial memory and which require hippocampal processing.

In those experiments where LTP was induced (Figure 1a, b) in response to theta burst stimulation, we found a small but statistically insignificant reduction in the extent of fEPSP potentiation with age. The incidence of LTP induction was, however, clearly reduced with age (Figure 1c). Whereas more than 80% of slices prepared from animals under 12 months of age underwent potentiation, fewer than 50% of slices from animals 18–30 months exhibited LTP. Our data are in accordance with previous studies in wild‐type rats and mice (see Burke & Barnes, 2006 for a review) and indicate that LTP is less likely to be induced in older animals and the progression of this deficit coincides with the age‐dependent cognitive decline revealed by our behavioural tests.

The size of fEPSPs recorded in the *stratum radiatum* of CA1 in response to identical intensity stimulation of SC‐AC fibres increased with age and this was accompanied by a significant reduction of the paired‐pulse ratio, suggesting that aging leads to an increase in release probability. Whilst other studies have found a reduction in synaptic strength with age (Barnes, Rao, Foster, & McNaughton, 1992; Rosenzweig, Rao, McNaughton, & Barnes, 1997), PPF of SC‐CA1 fEPSPs was similarly reduced in aged animals (Deupree et al., 1993) and frequency facilitation was absent in slices from aged rats compared to slices from animals 2 months old. This effect could be rescued by lowering the extracellular calcium/magnesium ratio (Landfield, Pitler, & Applegate, 1986), similarly suggesting an increase in release probability with age.

The increase in fEPSP size with age, and the concomitant reduction in the extent of PPF, was accompanied by an increase in the peak amplitude and initial slope of SyGCaMP2 fluorescence responses (Figure 2) and a slowing of recovery. This effect was maintained when postsynaptic transmission was blocked indicating a presynaptic locus. The peak amplitude of SyGCaMP2 fluorescence responses to electrical stimulation in single boutons from animals over 18 months old was significantly larger compared to those from animals <6 months of age. These effects were not due to changes in SyG:mCh expression levels since neither the density of SyGCaMP2 expressing boutons nor mCherry expression changed significantly with age (Figure 4a). These results are consistent with studies in which presynaptic calcium was assessed by loading SC fibres with membrane permeable dyes. Calcium responses to high‐frequency stimulation were elevated in slices from aged rats compared to those from younger rats (Tonkikh & Carlen, 2009), an effect reduced by the membrane permeable calcium chelator BAPTA‐AM. Increased calcium buffering recovered age‐dependent reductions in spatial memory and reversed the decrease in the extent of LTP associated with aging (Tonkikh et al., 2006).

It was possible to reverse these effects in aged mice by lowering the extracellular calcium concentration which reduces the inward driving force for calcium and lowers the probability of release. Conversely, raising extracellular calcium, which increases release probability, mimicked the effect of aging in slices obtained from young adults. These results indirectly suggest an increase in either the density, subunit composition or conductance of presynaptic calcium channels in aged animals. In postsynaptic compartments, calcium influx is similarly increased in the CA1 region of the hippocampus in aged rats. Expression of the Cav1.3 subunit of L‐type calcium channels is increased (Veng & Browning, 2002) and resting and stimulation‐induced calcium signalling through L‐type channels of CA1 pyramidal neurons is elevated compared to those from neurons recorded from slices obtained from younger animals (Thibault, Hadley, & Landfield, 2001; Thibault & Landfield, 1996). CA1 neurons from aged animals display an enhanced after‐hyperpolarization compared to those from young animals, also suggesting enhanced activation of calcium‐activated potassium channels. In addition, altered calcium buffering, extrusion and uptake (Martinez‐Serrano, Blanco, & Satrustegui, 1992; Raza et al., 2007), as well as mitochondrial dysfunction (Mattson & Liu, 2002) and a decline in Ca‐ATPase activity (Zaidi et al., 2003) contribute to calcium dysregulation. Although L‐type calcium channels are not expressed at presynaptic terminals, our results indicate that similar, age‐dependent processes may contribute to age‐dependent changes in calcium homeostasis in presynaptic terminals.

The SyG:mCh ratio is proportional to free calcium concentration allowing us to quantify absolute calcium in presynaptic terminals. We found that mean baseline calcium within CA1 increased with age from around 55 nM in animals under 6 months of age to around 140 nm in animals over 18 months old. These absolute values in slices obtained from younger animals were remarkably similar to those reported in aged guinea pigs using a membrane permeable form of the ratiometric calcium indicator Fura‐2 to measure calcium in SC fibres (Wu & Saggau, 1994). Moreover, the age‐dependent increase in resting calcium is in agreement with previous qualitative observations using a nonratiometric calcium indicator (Tonkikh et al., 2006). As animals aged, the proportion of slices with high resting levels of baseline presynaptic calcium increased. There is, therefore, a bimodal distribution indicating that during the aging process, the ability of neurons to homeostatically maintain calcium levels is lost leading to a change in overall resting baseline calcium state.

Ratiometric images of whole coronal brain slices suggested baseline presynaptic calcium concentration may also be raised in regions other than the hippocampus in aged mice (Figure 4d). In particular, the fibre tracts of the fornix, the major output pathway from the hippocampus, displayed a much higher baseline ratio in slices from older animals. Interestingly, SyG:mCh ratios were also high in the pyriform amygdaloid cortices and the retrosplenial cortex, an area of the brain that is involved in episodic memory, navigation, imagination and planning for the future (see Vann, Aggleton, & Maguire, 2009 for a review), cognitive functions that are affected during healthy aging.

## 4 EXPERIMENTAL PROCEDURES

### 4.1 Generation of SyGCaMP2‐mCherry mice

The generation, expression patterns and characteristics of SyG37 transgenic mice incorporating a SyGCaMP2‐mCherry transgene have already been described in detail (Al‐Osta et al., 2018). Briefly, SyGCaMP2‐mCherry was created by fusing mCherry to the C‐terminus of SyGCaMP2, which is, in turn, a fusion of GCaMP2 to the C‐terminus of synaptophysin‐1 (Dreosti, Odermatt, Dorostkar, & Lagnado, 2009). A Thy1‐2 promotor was used which produces founder‐dependent neuronal expression that appears from P7‐P10 onward and which is stable throughout adulthood (Al‐Osta et al., 2018). mCherry was used both to facilitate identification of synaptic boutons and as a reference to allow ratiometric quantification of expression levels and calibration of absolute calcium levels.

### 4.2 Behavioural experiments

Full details of the behavioural experiments can be found in the supplementary procedures.

### 4.3 Brain slice preparation

Animals of ages between 2 and 30 months were culled in accordance with Home Office regulations and local ethical approval and the brains quickly removed and placed in an ice cold artificial cerebrospinal fluid (aCSF) consisting of (in mM); 127 NaCl; 1.25 KH_2_PO_4_; 1.30 MgSO_4_.7H_2_O); 26 NaHCO_3_; 1.61 KCl; 10 glucose, equilibrated with 95% O_2_–5% CO_2_ to pH 7.4. Transverse hippocampal slices, 300 μm thick, were prepared using a vibratome (Dosaka EM, Japan) and equilibrated at room temperature in the same solution for at least one hour prior to experiments. The experimental procedures used for SyG37 imaging, electrophysiology and data analysis have previously been reported (Al‐Osta et al., 2018). Further details of methods associated with electrophysiological recordings and imaging can be found in the supplementary procedures.

### 4.4 Experimental design and statistical analysis

Experiments were carried out on brain slices from both male and female SyG37 and wild‐type (C57 Blk6) mice aged from 2 to 24 months. Data are presented as mean ± *SEM*. The numbers of replicates for experiments for each experiment are recorded in the text or in figure legends along with the levels of statistical significance. No more than two replicates of a single experiment were obtained from any one animal and replicates represent data from separate brain slices. Data were tested for normal distributions. The nonparametric Mann–Whitney U test (for unpaired data) or Wilcoxon test (for paired data) was used to test for statistical significance between two data sets. A 1‐way ANOVA with a Tukey post hoc comparison test was used to test for statistical differences between multiple groups of data that were normally distributed. A Kruskal–Wallis test with Dunn's multiple comparison was used for data that did not follow a normal distribution.

## CONFLICT OF INTEREST

None declared.

## AUTHOR CONTRIBUTIONS

DP carried out the majority of the experimental work, analysed data and helped to draft the manuscript. IA‐O contributed to the experimental work. AO carried out work to enable the quantification of calcium from the ratiometric calcium sensor. AE advised on and helped carry out and analyse the behavioural tasks. NH conceived the project, contributed to experimental work and data analysis, wrote software for analysis and drafted the manuscript. The authors have no conflicts of interest to declare.

## References

[acel13008-bib-0001] Al‐Osta, I. , Mucha, M. , Pereda, D. , Piqué‐Gili, M. , Okorocha, A. E. , Thomas, R. , & Hartell, N. A. (2018). Imaging calcium in hippocampal presynaptic terminals with a ratiometric calcium sensor in a novel transgenic mouse. Frontiers in Cellular Neuroscience, 12, 209 10.3389/fncel.2018.00209 30072872 PMC6060260

[acel13008-bib-0002] Barnes, C. A. (1979). Memory deficits associated with senescence: A neurophysiological and behavioral study in the rat. Journal of Comparative and Physiological Psychology, 93(1), 74–104. 10.1037/h0077579 221551

[acel13008-bib-0003] Barnes, C. A. , Rao, G. , Foster, T. C. , & McNaughton, B. L. (1992). Region‐specific age effects on AMPA sensitivity: Electrophysiological evidence for loss of synaptic contacts in hippocampal field CA1. Hippocampus, 2(4), 457–468. 10.1002/hipo.450020413 1284976

[acel13008-bib-0004] Barnes, C. A. , Rao, G. , & McNaughton, B. L. (1996). Functional integrity of NMDA‐dependent LTP induction mechanisms across the lifespan of F‐344 rats. Learning & Memory, 3(2–3), 124–137. 10.1101/lm.3.2-3.124 10456083

[acel13008-bib-0005] Barreto, G. , Huang, T. T. , & Giffard, R. G. (2010). Age‐related defects in sensorimotor activity, spatial learning, and memory in C57BL/6 mice. Journal of Neurosurgical Anesthesiology, 22(3), 214–219. 10.1097/ANA.0b013e3181d56c98 20479674 PMC2886171

[acel13008-bib-0006] Burke, S. N. , & Barnes, C. A. (2006). Neural plasticity in the ageing brain. Nature Reviews Neuroscience, 7(1), 30–40. 10.1038/nrn1809 16371948

[acel13008-bib-0007] Clark, R. E. , Zola, S. M. , & Squire, L. R. (2000). Impaired recognition memory in rats after damage to the hippocampus. Journal of Neuroscience, 20(23), 8853–8860. 10.1523/JNEUROSCI.20-23-08853.2000 11102494 PMC6773055

[acel13008-bib-0008] Deacon, R. M. , Croucher, A. , & Rawlins, J. N. (2002). Hippocampal cytotoxic lesion effects on species‐typical behaviours in mice. Behavioral Brain Research, 132(2), 203–213. 10.1016/S0166-4328(01)00401-6 11997150

[acel13008-bib-0009] Deacon, R. M. , Penny, C. , & Rawlins, J. N. (2003). Effects of medial prefrontal cortex cytotoxic lesions in mice. Behavioral Brain Research, 139(1–2), 139–155. 10.1016/S0166-4328(02)00225-5 12642185

[acel13008-bib-0010] Deacon, R. M. , & Rawlins, J. N. (2006). T‐maze alternation in the rodent. Nature Protocols, 1(1), 7–12. 10.1038/nprot.2006.2 17406205

[acel13008-bib-0011] Deupree, D. L. , Bradley, J. , & Turner, D. A. (1993). Age‐related alterations in potentiation in the CA1 region in F344 rats. Neurobiology of Aging, 14(3), 249–258. 10.1016/0197-4580(93)90009-Z 8321393

[acel13008-bib-0012] Deupree, D. L. , Turner, D. A. , & Watters, C. L. (1991). Spatial performance correlates with in vitro potentiation in young and aged Fischer 344 rats. Brain Research, 554(1–2), 1–9. 10.1016/0006-8993(91)90164-Q 1933293

[acel13008-bib-0013] Dreosti, E. , Odermatt, B. , Dorostkar, M. M. , & Lagnado, L. (2009). A genetically encoded reporter of synaptic activity in vivo. Nature Methods, 6(12), 883–889. 10.1038/nmeth.1399 19898484 PMC2859341

[acel13008-bib-0014] Ennaceur, A. , Michalikova, S. , van Rensburg, R. , & Chazot, P. L. (2008). Detailed analysis of the behavior and memory performance of middle‐aged male and female CD‐1 mice in a 3D maze. Behavioral Brain Research, 187(2), 312–326. 10.1016/j.bbr.2007.09.025 17983672

[acel13008-bib-0015] Forster, M. J. , Dubey, A. , Dawson, K. M. , Stutts, W. A. , Lal, H. , & Sohal, R. S. (1996). Age‐related losses of cognitive function and motor skills in mice are associated with oxidative protein damage in the brain. Proceedings of the National Academy of Sciences of the USA, 93(10), 4765–4769. 10.1073/pnas.93.10.4765 8643477 PMC39353

[acel13008-bib-0016] Forwood, S. E. , Winters, B. D. , & Bussey, T. J. (2005). Hippocampal lesions that abolish spatial maze performance spare object recognition memory at delays of up to 48 hours. Hippocampus, 15(3), 347–355. 10.1002/hipo.20059 15558543

[acel13008-bib-0017] Foster, T. C. , Barnes, C. A. , Rao, G. , & McNaughton, B. L. (1991). Increase in perforant path quantal size in aged F‐344 rats. Neurobiology of Aging, 12(5), 441–448. 10.1016/0197-4580(91)90071-Q 1770978

[acel13008-bib-0018] Fox, G. B. , Fan, L. , LeVasseur, R. A. , & Faden, A. I. (1998). Effect of traumatic brain injury on mouse spatial and nonspatial learning in the Barnes circular maze. Journal of Neurotrauma, 15(12), 1037–1046. 10.1089/neu.1998.15.1037 9872460

[acel13008-bib-0019] Landfield, P. W. , Pitler, T. A. , & Applegate, M. D. (1986). The effects of high Mg2+‐to‐Ca2+ ratios on frequency potentiation in hippocampal slices of young and aged rats. Journal of Neurophysiology, 56(3), 797–811. 10.1152/jn.1986.56.3.797 3783221

[acel13008-bib-0020] Martinez‐Serrano, A. , Blanco, P. , & Satrustegui, J. (1992). Calcium binding to the cytosol and calcium extrusion mechanisms in intact synaptosomes and their alterations with aging. Journal of Biological Chemistry, 267(7), 4672–4679. 1531657

[acel13008-bib-0021] Mattson, M. P. , & Liu, D. (2002). Energetics and oxidative stress in synaptic plasticity and neurodegenerative disorders. Neuromolecular Med, 2(2), 215–231. 10.1385/NMM:2:2:215 12428812

[acel13008-bib-0022] Mizoguchi, K. , Shoji, H. , Tanaka, Y. , Maruyama, W. , & Tabira, T. (2009). Age‐related spatial working memory impairment is caused by prefrontal cortical dopaminergic dysfunction in rats. Neuroscience, 162(4), 1192–1201. 10.1016/j.neuroscience.2009.05.023 19463906

[acel13008-bib-0023] Mongillo, G. , Barak, O. , & Tsodyks, M. (2008). Synaptic theory of working memory. Science, 319(5869), 1543–1546. 18339943 10.1126/science.1150769

[acel13008-bib-0024] Moore, C. I. , Browning, M. D. , & Rose, G. M. (1993). Hippocampal plasticity induced by primed burst, but not long‐term potentiation, stimulation is impaired in area CA1 of aged Fischer 344 rats. Hippocampus, 3(1), 57–66. 10.1002/hipo.450030106 8364683

[acel13008-bib-0025] Morrison, J. H. , & Baxter, M. G. (2012). The ageing cortical synapse: Hallmarks and implications for cognitive decline. Nature Reviews Neuroscience, 13(4), 240–250. 10.1038/nrn3200 22395804 PMC3592200

[acel13008-bib-0026] Norris, C. M. , Korol, D. L. , & Foster, T. C. (1996). Increased susceptibility to induction of long‐term depression and long‐term potentiation reversal during aging. Journal of Neuroscience, 16(17), 5382–5392. 10.1523/JNEUROSCI.16-17-05382.1996 8757251 PMC6578896

[acel13008-bib-0027] Rapp, P. R. , & Gallagher, M. (1996). Preserved neuron number in the hippocampus of aged rats with spatial learning deficits. Proceedings of the National Academy of Sciences of the USA, 93(18), 9926–9930. 10.1073/pnas.93.18.9926 8790433 PMC38531

[acel13008-bib-0028] Raza, M. , Deshpande, L. S. , Blair, R. E. , Carter, D. S. , Sombati, S. , & DeLorenzo, R. J. (2007). Aging is associated with elevated intracellular calcium levels and altered calcium homeostatic mechanisms in hippocampal neurons. Neuroscience Letters, 418(1), 77–81. 10.1016/j.neulet.2007.03.005 17374449 PMC2094130

[acel13008-bib-0029] Robitsek, R. J. , Fortin, N. J. , Koh, M. T. , Gallagher, M. , & Eichenbaum, H. (2008). Cognitive aging: A common decline of episodic recollection and spatial memory in rats. Journal of Neuroscience, 28(36), 8945–8954. 10.1523/JNEUROSCI.1893-08.2008 18768688 PMC2585597

[acel13008-bib-0030] Rosenzweig, E. S. , Rao, G. , McNaughton, B. L. , & Barnes, C. A. (1997). Role of temporal summation in age‐related long‐term potentiation‐induction deficits. Hippocampus, 7(5), 549–558. 10.1002/(SICI)1098-1063(1997)7:5<549:AID-HIPO10>3.0.CO;2-0 9347351

[acel13008-bib-0031] Thibault, O. , Hadley, R. , & Landfield, P. W. (2001). Elevated postsynaptic [Ca2+]i and L‐type calcium channel activity in aged hippocampal neurons: Relationship to impaired synaptic plasticity. Journal of Neuroscience, 21(24), 9744–9756. 11739583 10.1523/JNEUROSCI.21-24-09744.2001 PMC6763040

[acel13008-bib-0032] Thibault, O. , & Landfield, P. W. (1996). Increase in single L‐type calcium channels in hippocampal neurons during aging. Science, 272(5264), 1017–1020. 8638124 10.1126/science.272.5264.1017

[acel13008-bib-0033] Tonkikh, A. A. , & Carlen, P. L. (2009). Impaired presynaptic cytosolic and mitochondrial calcium dynamics in aged compared to young adult hippocampal CA1 synapses ameliorated by calcium chelation. Neuroscience, 159(4), 1300–1308. 10.1016/j.neuroscience.2008.12.057 19215725

[acel13008-bib-0034] Tonkikh, A. , Janus, C. , El‐Beheiry, H. , Pennefather, P. S. , Samoilova, M. , McDonald, P. , … Carlen, P. L. (2006). Calcium chelation improves spatial learning and synaptic plasticity in aged rats. Experimental Neurology, 197(2), 291–300. 10.1016/j.expneurol.2005.06.014 16039651

[acel13008-bib-0035] Vann, S. D. , Aggleton, J. P. , & Maguire, E. A. (2009). What does the retrosplenial cortex do? Nature Reviews Neuroscience, 10(11), 792–802. 10.1038/nrn2733 19812579

[acel13008-bib-0036] Veng, L. M. , & Browning, M. D. (2002). Regionally selective alterations in expression of the alpha(1D) subunit (Ca(v)1.3) of L‐type calcium channels in the hippocampus of aged rats. Brain Research. Molecular Brain Research, 107(2), 120–127. 12425941 10.1016/s0169-328x(02)00453-9

[acel13008-bib-0037] Vinters, H. V. (2015). Emerging concepts in Alzheimer's disease. Annual Review of Pathology: Mechanisms of Disease, 10, 291–319. 10.1146/annurev-pathol-020712-163927 25387055

[acel13008-bib-0038] Wu, L. G. , & Saggau, P. (1994). Presynaptic calcium is increased during normal synaptic transmission and paired‐pulse facilitation, but not in long‐term potentiation in area CA1 of hippocampus. Journal of Neuroscience, 14, 645–654. 10.1523/JNEUROSCI.14-02-00645.1994 7905515 PMC6576817

[acel13008-bib-0039] Zaidi, A. , Barron, L. , Sharov, V. S. , Schoneich, C. , Michaelis, E. K. , & Michaelis, M. L. (2003). Oxidative inactivation of purified plasma membrane Ca2+‐ATPase by hydrogen peroxide and protection by calmodulin. Biochemistry, 42(41), 12001–12010. 10.1021/bi034565u 14556631

