# Supplementary figures and images for: Changes in presynaptic calcium signalling accompany age‐related deficits in hippocampal LTP and cognitive impairment
Source: Aging Cell. 2019 Jul 16;18(5):e13008. doi: 10.1111/acel.13008 (PMC6718530; doi:10.1111/acel.13008)

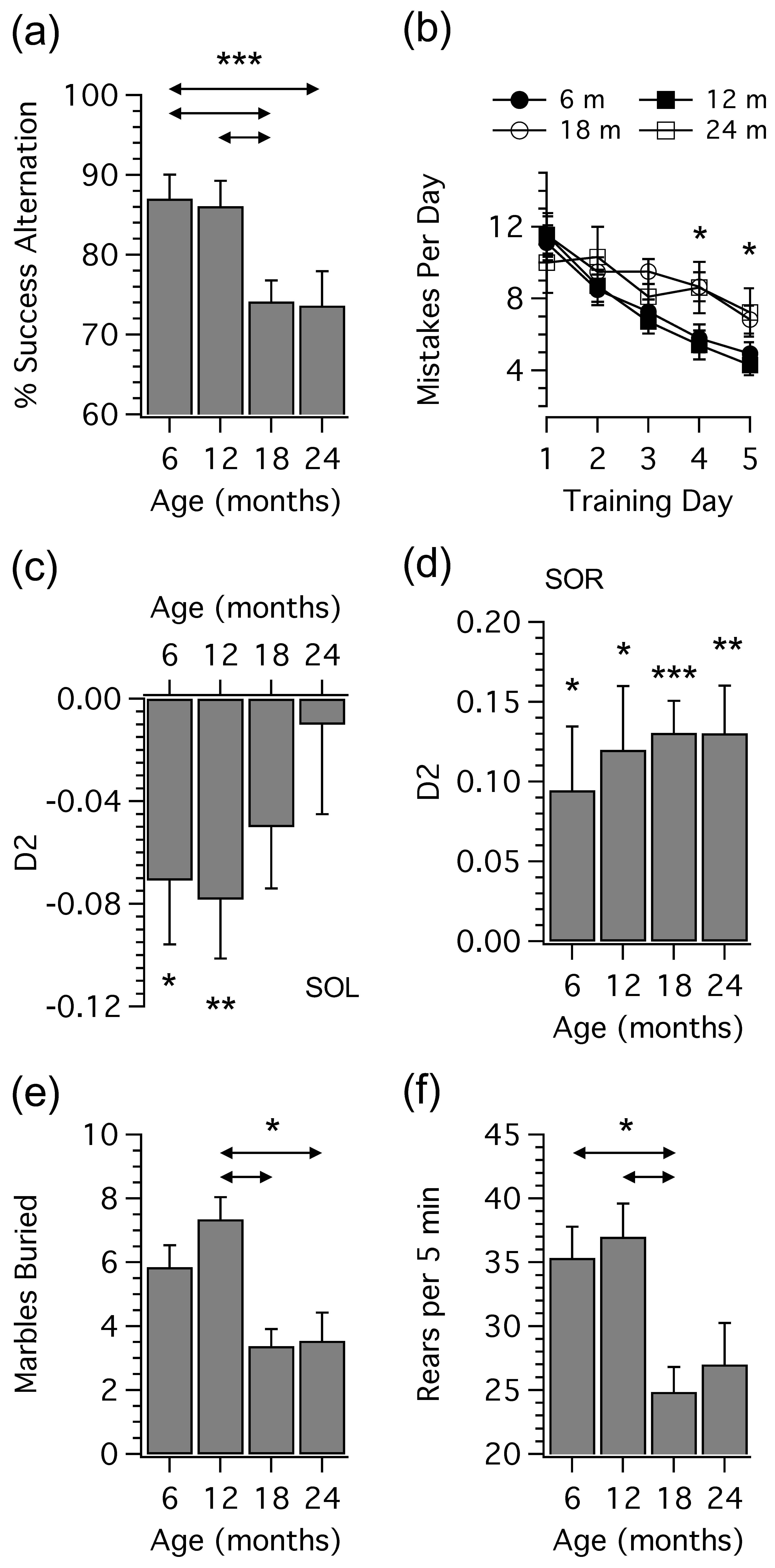

Supplement: Supplementary file 1 [file ACEL-18-e13008-s001.tiff]

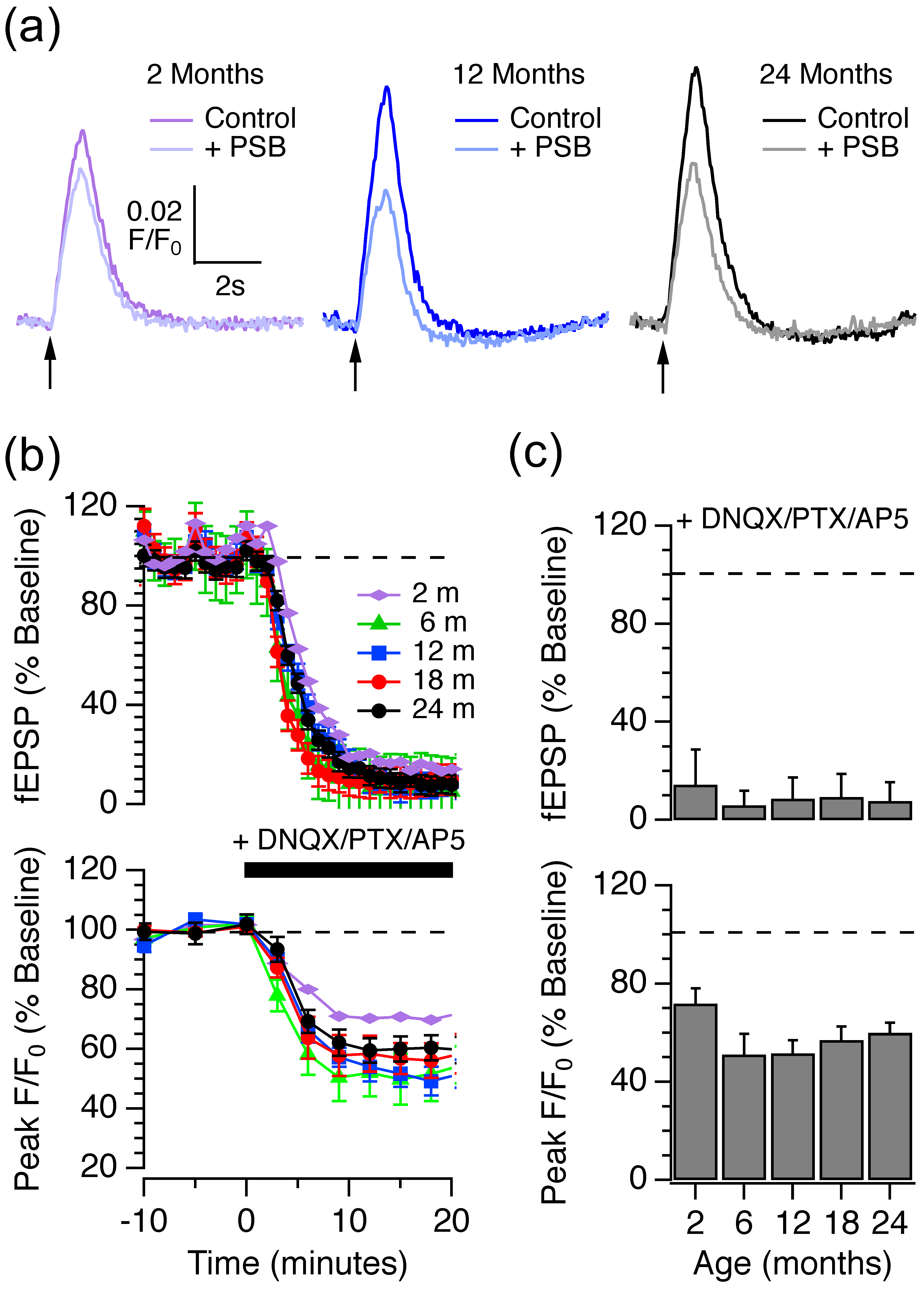

Supplement: Supplementary file 2 [file ACEL-18-e13008-s002.tiff]
