# Supplementary material for: Changes in presynaptic calcium signalling accompany age‐related deficits in hippocampal LTP and cognitive impairment
Source: Aging Cell. 2019 Jul 16;18(5):e13008. doi: 10.1111/acel.13008 (PMC6718530; doi:10.1111/acel.13008)
Supplement: Supplementary file 3 [file ACEL-18-e13008-s003.docx]

**Supplemental Experimental Procedures**

*Behavioural experiments*

In order to establish over what age range hippocampal function becomes impaired in mice incorporating the SyGCaMP2-mCherry transgene, groups of male and female mice aged in groups of 6, 12, 18 and 24 months underwent a series of different cognitive behavioural tasks which have previously been shown to require various degrees of hippocampal processing. Animals were housed and maintained in single-sided cages with environmental enrichment, a 12-h light–dark cycle with food and water available *ad libitum*. Behavioural experiments were performed during the light phase of the light-dark cycle in accordance with institutional and national guidelines. Mice were housed in groups of 3–6 litter mates per cage. Groups sizes, in terms of total animals and proportions of male/female were 6 months, n=20 (13/7), 12 months, n=23 (14/9), 18 months, n=29 (14/15) and 24 months, n=11 (7/4). The reduced number of animals at 24 months was due to natural mortality. Any mice that showed signs of ill health were retired from experiments. Prior to experiments, mice were habituated to the behavioural room for an hour every day for a week before starting the experiments and subjected to handling by researchers. The experimental room was sound-proofed and maintained at 21 ± 2 ˚C at the same light intensity. Behavioural apparatus were thoroughly cleaned with distilled water and dried before each use. With the exception of the marble burying test, experiments were recorded and analysed blind offline.

A black acrylic T-maze arena (Deacon, 2006) was set up to evaluate spontaneous alternation behaviour. Each mouse was carefully placed in the start area at the far side of the long arm, with a central partition in place to better allow the mouse to select one of the two open goal arms to the sides. Once the mouse had chosen and entered an arm, it was confined there by a sliding door for 30 seconds. After that time, the central partition was removed, the sliding door raised and the animal replaced in the start position facing away from the goal arms to allow it to choose again between the open goal arms. A successful alternation was called when the animal entered the arm not chosen during the first phase. Every mouse was subjected to a 10-trial block scheduled as two experiments per day over five consecutive days.

Open field activity was recorded in a transparent-walled, white-floored, plastic, 50 cm square open field arena with a wall height of 60 cm into which each mouse was placed at the same spot, facing the wall. Its activity was recorded for 5 min. Parameters analysed included the number of lateral transitions between sectors drawn onto the white floor, as well as the number of rearing events, defined as the animal standing on its rear legs and leaning against the wall.

In a marble burying test, mice were introduced to a clean housing cage filled with 5 cm of clean bedding on which 9, green, glass marbles were placed in a 3 × 3 equidistant arrangement. Animals were removed after a 30-min exploration period and the 9 marbles classified as buried, half buried or un-buried.

A standard, white, acrylic Barnes maze (Sunyer, Patil, Höger, & Lubec, 2007) was set up to evaluate navigational/spatial memory in our cohorts. Each mouse was carefully placed in a high walled box in the middle of an elevated circular maze (92 cm diameter) where 20 holes (5 cm diameter; 7.5 cm between holes) had been drilled 2 cm from away from the perimeter of the maze. One of the holes led to a black acrylic escape compartment fixed underneath where the animal could find refuge from the white open arena. After ten seconds in the box, it was removed and mice were allowed to explore the arena for 1 minute. Every mouse was subjected to a 20-trial block scheduled as four experiments a day over five consecutive days.

A continual trial apparatus was set up to evaluate spontaneous object location (SOL) and spontaneous object recognition (SOR) memory in our cohorts (see Ameen-Ali, Eacott, & Easton, 2012 for details). Briefly, the square apparatus was divided into two different sized E-shaped areas, a holding chamber and an object chamber, separated by guillotine doors that could be opened and closed independently by the researcher. An extended habituation period lasting three days was allowed during which the mice were progressively exposed to both areas of the apparatus and trained to shuttle between them, as well as to a range of objects of various sizes, colours and textures, similar to those employed during the experiments. Mice were introduced to the apparatus initially in pairs and then individually. A reward was not used for training. Each mouse was then subjected to two different paradigms with a three-week break in between.

In the SOR task, two identical copies of the same object were placed in the object area and the mouse was allowed to interact with them for two minutes (exploration phase), after which the lateral guillotine doors were opened and the animal shuttled back to the holding area for one minute, while one of the copies of the object was replaced for a different one. Then the central guillotine door was opened and the mouse shuttled to the object area, where it was allowed to interact with both objects for two more minutes (test phase). Each mouse was subjected to sixteen consecutive, different trials within a single session, using sixteen different random object sets, with the position of the new object changing between mice in a semi-random sequence. Object discrimination (D2) in each test phase was measured by calculating the difference in exploration time for the novel object minus that for the familiar object divided by the total exploration time (Ennaceur, 1998). In the SOL task, the protocol was the same but the exploration and test phase were inverted, with the mice being exposed to identical objects during test and exploration phases but with novelty now being determined by the location of an object in a place it had not previously appeared in.

*Electrophysiological recordings*

For LTP experiments, pairs of stimuli were delivered to the *stratum radiatum* (*s. radiatum*) between CA3 and CA1 with an aCSF filled patch pipette at an interval of 50 ms every 10 seconds to assess the level of paired pulse facilitation of fEPSPs recorded in the *s. radiatum* of CA1. Once a stable baseline response had been established, theta burst stimulation was applied which consisted of 20 trains of 4 stimuli delivered at 100 Hz, repeated at an interval of 350 ms. The extent and incidence of LTP within each age group of animals was then assessed. These experiments were performed at 36˚C.

*Image Analysis*

Image analysis was also carried out as previously described (Al-Osta et al., 2018). For wide-field epifluorescence measurements, stacks of interleaved SyGCaMP2 and mCherry images were separated and regions of interest (ROIs) were placed over defined areas of the slices and changes in absolute fluorescence measured over time. The time constants for photobleaching were measured and used to remove the effects of bleaching on stimulated responses. The mean fluorescence values for each bleach compensated ROI prior to electrical stimulation were calculated (F_0_) and the absolute fluorescence expressed as a proportion of this (F/F_0_). The last measurement before stimulation was taken as time zero and data were plotted relative to the onset of stimulation. Measurements of the peak amplitude of the responses, time to peak, the initial slope, time constant of decay and area under the curve were made from each image stack and then these values pooled from several experiments and plotted against time.

*Image Stitching*

Images of whole brain sections were taken using an epifluorescence microscope equipped with an sCMOS camera (Prime; Photometrics) with a x4 air immersion objective (NA 0.2). Slices were placed on a section of insert designed for use with organotypic cultures and perfused slowly with oxygenated aCSF during the imaging process to minimise movement. Arrays of images were flattened and stitched together as previously described (Al-Osta et al., 2018). Green (SyGCaMP2) and red (mCherry) images of slices prepared from animals of each age range were displayed with identical brightness settings using Igor Pro. The ratio of green to red images was calculated by first background subtracting mean fluorescence values measured from WT brain slices and then dividing green images by red images. The ratio images were displayed using a look up table with identical brightness settings. The absolute ratio is somewhat arbitrary as it depends upon the relative intensities of light used to excite SyGCaMP2 and mCherry.

*Single Bouton Imaging*

Images of single boutons were obtained using a Zeiss MP5 multiphoton microscope (Carl Zeiss Microscopy GmbH, Jena, Germany) using an excitation wavelength of 920 nm and a 20 x objective (0.8 NA) with an optical zoom of 3. Stacks of 80 images comprising 512 pixels by 64 lines were collected at intervals of 110 ms. Single bouton responses were analysed using Igor Pro with both custom routines and a package of semi-automated routines designed for measuring calcium responses from presynaptic boutons (see Dorostkar, Dreosti, Odermatt, & Lagnado, 2010 for a detailed description). Regions of interest were identified either manually or by thresholding background subtracted images constructed by averaging over time each stack of 100 images per stimulus condition. Averaged images were transformed using the Laplace operator and then a multiple of the standard deviation (SD) of all the pixel values in the Laplace operator used as a threshold. Changes in fluorescence over time from each identified region of interest were then converted into F/F_0_ values and each plotted as single lines to create a 2D image illustrating stimulus dependent changes in fluorescence over time. A pseudo-colour look-up table was applied to highlight effects. A semi-automated hierarchical clustering analysis was then used to categorise each region of interest as either responding to stimulation or not responding. The results of this clustering were verified manually. The number of regions of interest per stimulus condition that responded to stimulation was then recorded as a proportion of the total number of regions identified. F/F_0_ responses for responding regions were pooled together to create an average response for responders under each stimulus condition. To illustrate the positions of responding and non-responding puncta, green and red dots were plotted on the original images used for thresholding centred at the point of maximum intensity for each region of interest.

Supplemental Figure Legends

**Figure S1**

*Age-dependent changes in hippocampal related behaviour in SyG37 mice.* (a) The mean percentage of successful spontaneous alternations for the T-maze test are shown along with standard errors of the mean. Group sizes were 20, 23, 29 and 11 mice in ascending age order. There was a significant effect of age on the percentage of successful alternation (Kruskal-Wallace; P<0.001). The horizontal arrows indicate where statistical differences between individual age groups occurred (Dunne-Holland-Wolfe test; P<0.05). The effects of age on the performance of mice in the Barnes maze are shown in panel (b). Asterisks indicate where statistically significant differences between adult (6-12 months old) and aged (18-24 months old) mice were observed. The effects of age on SOL (c) and SOR (d) are shown. Asterisk indicate where performance was significantly different from chance (* P< 0.05; ** P< 0.01; *** P<0.001). Panels (e) and (f) illustrate the effects of rearing in an open field test and marbles buried in the same aged cohorts of mice. Data are also shown as means ± standard errors of the mean. Age had a statistically significant effect on each test (P<0.0005; Kruskal-Wallace Test; P<0.05). Horizontal arrows indicate where statistical differences between individual age groups occurred (Dunne-Holland-Wolfe test; P<0.05).

**Figure S2**

*Age-dependent changes in the relative contributions of directly activated and synaptically activated presynaptic terminals to SyGCaMP2 responses.* (a) Examples of SyGCaMP2 responses to 10Hz stimulation of the SC pathway to CA1 before (darker traces) and after (lighter traces) applications of a cocktail of post-synaptic receptor blockers consisting of DNQX, PTX and AP5. (b) Pooled data showing the effect of washing on DNQX, PTX and AP5 on fEPSPs (upper panel) and SyGCaMP2 responses (lower panel) at each of the age ranges shown. (c) Data illustrating the final effect of these blockers on fEPSPs (top) and fluorescent responses (bottom). Whilst the addition of PSBs produced a significant reduction in the size of fEPSPs and peak SyGCaMP2 responses, there was no differential effect of age (Kruskal-Wallis; P> 0.3). The number of replicates in 6, 12, 18 and 24-month age groups were 20, 17, 12 and 16 respectively.

Figure S1

Figure S2

Al-Osta, I., Mucha, M., Pereda, D., Piqué-Gili, M., Okorocha, A. E., Thomas, R., & Hartell, N. A. (2018). Imaging Calcium in Hippocampal Presynaptic Terminals With a Ratiometric Calcium Sensor in a Novel Transgenic Mouse. *Front Cell Neurosci, 12*, 209.

Ameen-Ali, K. E., Eacott, M. J., & Easton, A. (2012). A new behavioural apparatus to reduce animal numbers in multiple types of spontaneous object recognition paradigms in rats. *J Neurosci Methods, 211*(1), 66-76. doi:10.1016/j.jneumeth.2012.08.006

Deacon, R. M. (2006). Burrowing in rodents: a sensitive method for detecting behavioral dysfunction. *Nat Protoc, 1*(1), 118-121. doi:10.1038/nprot.2006.19

Dorostkar, M. M., Dreosti, E., Odermatt, B., & Lagnado, L. (2010). Computational processing of optical measurements of neuronal and synaptic activity in networks. *J Neurosci Methods, 188*(1), 141-150. doi:10.1016/j.jneumeth.2010.01.033

Ennaceur, A. (1998). Effects of lesions of the Substantia Innominata/Ventral Pallidum, globus pallidus and medial septum on rat's performance in object-recognition and radial-maze tasks: physostigmine and amphetamine treatments. *Pharmacol Res, 38*(4), 251-263. doi:10.1006/phrs.1998.0361

Nadkarni, S., Bartol, T. M., Sejnowski, T. J., & Levine, H. (2010). Modelling vesicular release at hippocampal synapses. *PLoS Comput Biol, 6*(11), e1000983. doi:10.1371/journal.pcbi.1000983

Sunyer, B., Patil, S., Höger, H., & Lubec, G. (2007). Barnes maze, a useful task to assess spatial reference memory in the mice.
